# Expeditious Mode of Applying Moxa

**Published:** 1831-01-01

**Authors:** 


### Expeditious Mode of applying Moxa. By *M. Potet.*

The author of this paper, in Broussais' Journal, proposes, and has put in practice, a substitute for the moxa, which he affirms to be less painful and more expeditious than the Chinese procedure. It is neither more nor less than laying a train of common gunpowder on the part, or rather on a plate under the part, and blowing it up by a match. The process is quick as lightning, and the extent as well as depth of the eschar may be regulated by the quantity of gunpowder employed. Four cases are related in illustration. The first was a case of hemiplegia in an aged female. M. Potet laid a train of gunpowder on a plate under the paralyzed leg, about an inch broad at one end, and gradually diminishing to a point at the other. The limb was held about six inches above the train when fired. The patient seemed electrified, and drew the paralytic leg out of the assistant's hands. The power of motion also returned in the arm, and her mouth returned to its natural position. The reporter was not a little surprised at this event. The scorched part was merely dressed with cabbage leaves — suppuration became copious and continued a month, by which time motion was re-established in the paralytic side, and continues so till the present time, three years after the burning. The hemiplegia had been of a month's standing before the above remedy was employed.

The second case was one of paralysis of the right arm, of six months' duration. The gunpowder moxa was applied from the wrist to the elbow. Motion was instantaneously restored. The suppuration was very trifling. Case three was a female who was seized with complete hemiplegia on the 2d of April, 1830. M. Potet applied the explosive moxa from the wrist to the elbow. Motion was immediately restored, as was also speech, which had been considerably affected. She only kept her bed a few days, but the suppuration continued for nearly a month. So much muscular power continued in the affected side that she was able to pursue her domestic avocation, which was spinning.

The last case was that of a man who was grievously afflicted with sciatica of both the lower extremities, and who had been given up by a quack after all his money had been drained. A superficial moxa was applied twice from the right ankle up to the top of the thigh. He proposed to apply a third one, but was refused. On the third day this man could walk with the aid of a stick; and by the end of a fortnight he was able to take to his work.

Should the fulminating moxa be found as useful as the less alarming Asiatic process, it will certainly be much more manageable. But we fear that there is something so repugnant to the feelings of patients in this remedy, that it will never come into general use, however beneficial in itself. The fatal accidents, too, which have resulted from the counter-irritations of St. John Long, will prove an obstacle to the judicious and necessary employment of such measures, for many years to come. The abuse of a remedy will always curtail its use; and the recent exposure of quackeries will prove a considerable check on legitimate and scientific remedies.

